# Eco-friendly resolution of spectrally overlapping signals of a combined triple-action over-the-counter pharmaceutical formulation for symptomatic management of COVID-19 pandemic: application to content uniformity testing

**DOI:** 10.1186/s13065-022-00868-0

**Published:** 2022-10-03

**Authors:** Hoda M. Marzouk, Engy A. Ibrahim, Maha A. Hegazy, Samah S. Saad

**Affiliations:** 1 Analytical Chemistry Department, Faculty of Pharmacy, Cairo University, Kasr Al-Aini Street, Cairo, 11562 Egypt; 2 Pharmaceutical Analytical Chemistry Department, College of Pharmaceutical Sciences and Drug Manufacturing, Misr University for Science & Technology, 6th of October City, Giza Egypt

**Keywords:** Aspirin, Content uniformity testing, Diphenhydramine, D^1^ method, DWRS method, DD-RD method, Greenness assessment, Paracetamol, Spectrophotometry

## Abstract

Currently, all researchers are concentrating their efforts on countering the COVID-19 pandemic. The majority of patients are managed at home, according to recent statistics. An OTC triple action combination comprising paracetamol (PAR), aspirin (ASP), and diphenhydramine (DIPH) is commonly given for pain relief, fever control, and as a night-time sleep aid. This combination is currently recommended for COVID-19 patients as part of symptomatic treatment and management. In this work, three smart, simple, accurate, eco-friendly, and cost-effective spectrophotometric methods are developed for simultaneous determination of PAR, ASP, and DIPH in their combined over-the-counter caplet dosage form without any prior separation steps. The first method is the first derivative spectrophotometry (D^1^) which determined PAR at 259.7 nm. The second one is the dual-wavelength in ratio spectra (DWRS) for determination of ASP at 214.1 and 220.1 nm after using 10.0 μg/mL of PAR as a divisor, where PAR was a constant, and the wavelengths difference equal to zero for DIPH. The third method is the double divisor-ratio difference spectrophotometric one (DD-RD) which was based on using the sum of 15.0 µg/mL of each of PAR and ASP as a double divisor, and the difference in amplitudes was measured at two wavelengths ∆P_(214.5–226.0)_ for determination of DIPH. The developed methods have been validated as per ICH guidelines. Furthermore, the three suggested methods were employed successfully to assay marketed pharmaceutical formulation and to investigate the content uniformity of the dosage units in accordance with the United States Pharmacopeia's guidelines. Finally, the greenness profile of the proposed methods was assessed and compared with the reported method using the analytical eco-scale system, national environmental method index (NEMI), green analytical procedure index (GAPI), and analytical greenness (AGREE) metric. The results from the proposed methods statistically agreed with those obtained by the reported one, with no significant differences in accuracy and precision.

**Graphical Abstract:**

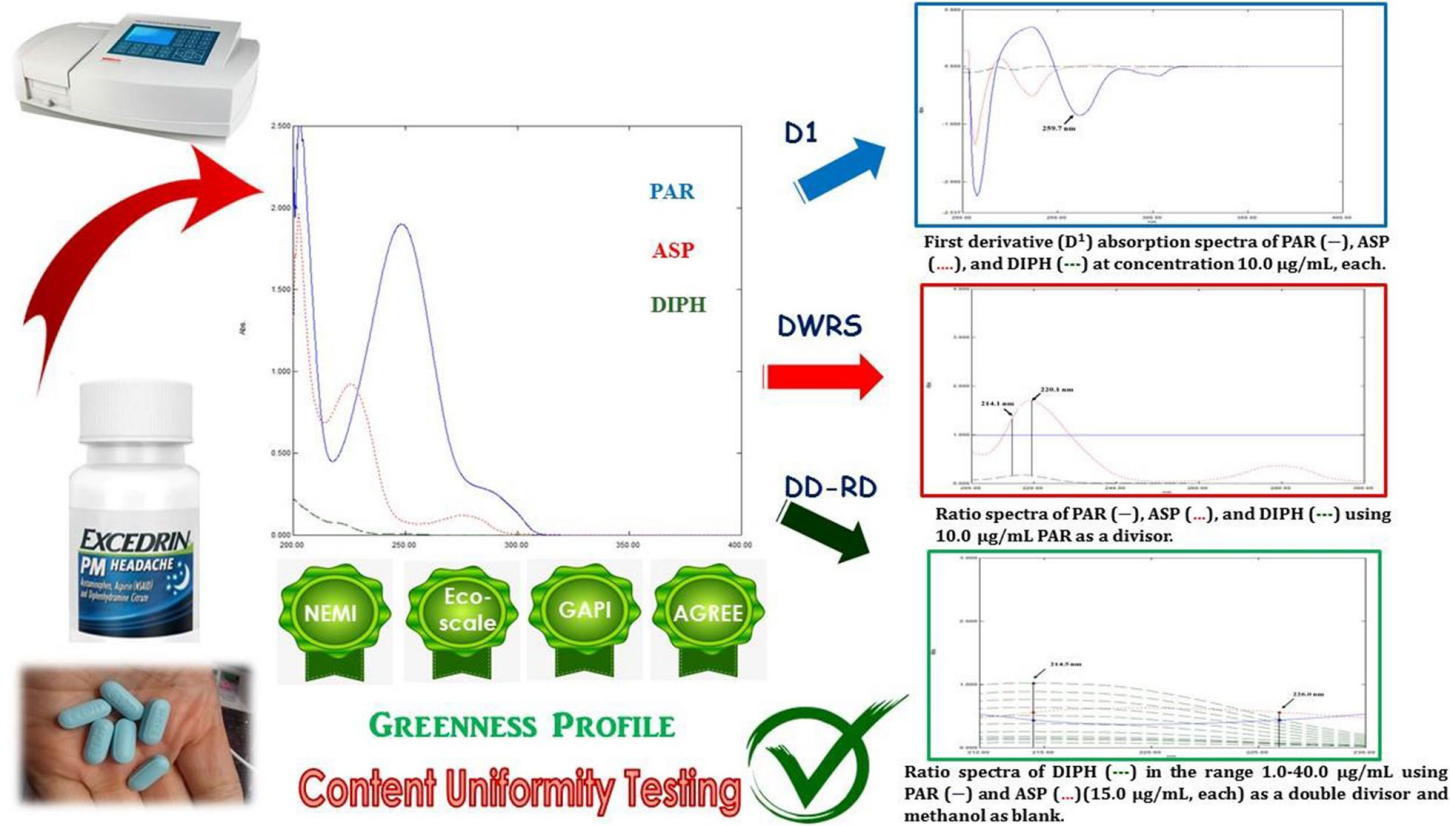

## Introduction

Spectrophotometric technique has perceived a significant progress in developing rapid and effective resolution procedures for spectrally overlapping signals of drug combinations. It provides comparable reliability and accuracy to different hyphenated chromatographic techniques without the requirement for complex computer systems, highly skilled analysts, tedious analytical process, or costly instrument set up. Accordingly, spectrophotometry has drawn the attention as a time saving, cost-effective and eco-friendly potential candidate where minimal energy and harmful solvents are used with decreased waste generation [1, 2].

Regulatory guidance and pharmacopeia provide a series of routine quality control procedures to guarantee that finished pharmaceutical formulations meet the required specifications. One of these procedures is content uniformity testing, which involves selecting different dosage units at random and analyzing each one separately using a relevant analytical method [3]. It represents the active drug's uniform distribution within a production batch and sheds light on the magnitude of variations between dose units. Moreover, it is also regarded as an important requirement before performing a bioequivalence study to ensure that study subjects get the active component at a dosage that does not deviate from the labelled quantity by more than the defined value [4, 5]. However, employing chromatographic methods for such testing, on regular basis, consumes a lot of resources and time. Hence, when it comes to the launch of new pharmaceutical formulations, a simple, rapid, and reliable analytical method is always preferred, while environmental, health and safety considerations shall always be taken into account.

**Fig. 1:**
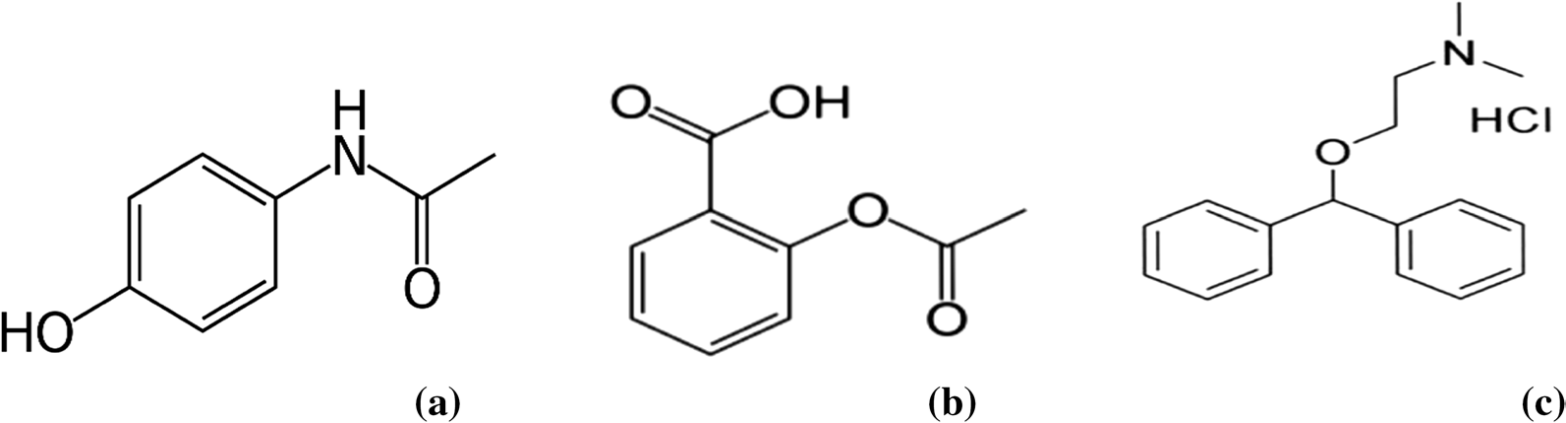
Chemical structure of **a** Paracetamol, **b** Aspirin, **c** Diphenhydramine Hydrochloride

Oral pharmaceutical formulation containing paracetamol (PAR), aspirin (ASP) and diphenyhyramine (DIPH) is commonly available as over-the-counter (OTC) to tackle night-time headache pain. PAR, N-(4-Hydroxyphenyl) acetamide, acts as an antipyretic and analgesic agent [6]. ASP, 2-(Acetyloxy)benzoic acid, is a non-selective cyclo-oxygenase inhibitor; antipyretic; analgesic; and anti-inflammatory. DIPH, 2-(Diphenylmethoxy)-N,N-dimethylethylamine hydrochloride, is a first-generation antihistamine [6]. The relevance of the listed medications arises from recent studies on the possibility of using them as an add-on treatment for COVID-19 patients, particularly those who are home-treated and have moderate cases [7–9]. The chemical structures of PAR, ASP and DIPH are shown in Fig. 1. According to the literature in hands, only HPLC–DAD and HPTLC-densitometric methods have been recently reported to determine the cited drugs in the pharmaceutical formulation [10]. However, it requires costly instrumentation, expert operators, lengthy analysis time and consume more hazardous solvents.

Analysts recently tried to use a competent methodological approach that is more eco-friendly and sustainable in order to protect our environment from chemical hazards [11–15]. Accordingly, this research aims to establish simple, reliable, fast, eco-friendly, and low-cost analytical methodology for determination of PAR, ASP, and DIPH in their new co-formulated ternary mixture. This was accomplished through applying smart and simple spectrophotometric methods, for the first time, namely; first derivative spectrophotometric method (D^1^), dual wavelength in ratio spectra spectrophotometric method (DWRS), and double divisor-ratio difference spectrophotometric method (DD-RD) for resolving the spectrally overlapping mixture. The developed validated methods were exploited for assaying the cited drugs in their commercial combined pharmaceutical formulation as well as assessing their content uniformity. The presented spectrophotometric approach offered simple, cost-effective and reliable platform for routine quality control assessment, without preliminary separation, in pharmaceutical industry.

## Experimental

### Apparatus and chemicals

Spectrophotometric measurements were carried out on a UV-1650 PC double beam UV–visible spectrophotometer (Shimadzu, Kyoto, Japan), using 1 cm quartz cell. Data analysis and manipulation was performed using UV Probe software version 2.51. The scanning speed was 2800 nm/min, and the spectral slit width was 2 nm.

Pure standard sample of PAR was supplied by El Nasr Pharmaceutical Co. (Cairo, Egypt), whereas ASP was provided by Al-Gomhoria Chem. Co. (Cairo, Egypt). Their purities were determined and found to be 99.80 ± 0.50% for PAR [6] and 100.30 ± 1.87% for ASP [16], applying their official methods. DIPH pure sample as hydrochloride salt was obtained from Wanbury Ltd. Co. (India). Its purity was found to be 100.54 ± 0.83% according to BP method [6].

Excedrin^®^ PM Headache caplets (Batch Number; 46172679) were manufactured by GlaxoSmithKline, USA. Each caplet claimed to contain 250 mg PAR, 250 mg ASP, and 38 mg DIPH as citrate salt and was obtained from the local market. Methanol of HPLC-grade was used as solvent during measurements (Fisher Scientific, UK).

### Stock and working standard solutions

Stock standard solutions of PAR, ASP, and DIPH (100.0 µg/mL) were prepared, separately, by transferring an accurate weight of each pure sample to 100-mL volumetric flask and using methanol as a solvent. Further dilutions were carried out by withdrawing different volumes from the relevant stock standard solution and transferred into a 10-mL volumetric flask, then diluted to the mark with methanol.

### Procedure

In three individual sets of 10-mL volumetric flasks, different standard solutions were prepared, over a concentration range of 3.0–40.0 µg/mL for PAR, 4.0–40.0 µg/mL for ASP and 1.0–40.0 µg/mL for DIPH in methanol. The absorption spectra of the prepared solutions were UV scanned at 200.0–400.0 nm. A set of five laboratory prepared mixtures of PAR, ASP and DIPH was prepared in different ratios and measured against methanol as blank. The absorption spectra were recorded, and subsequent manipulations were performed for the determination of each drug separately as follows:

#### First derivative spectrophotometric method (D^1^) for PAR determination

The absorption spectra of PAR were subjected to *D*^*1*^ calculation using scaling factor (10) and Δλ = 4 and the first derivative spectra of PAR were obtained. The peak amplitudes were recorded at 259.7 nm and plotted *versus* the corresponding concentration to construct the calibration curve. The regression parameters were evaluated.

#### Dual wavelength in ratio spectra (DWRS) for ASP determination

The absorption spectra of ASP were divided by a spectrum of 10.0 µg/mL PAR. The amplitudes of the ratio spectra were recorded at 214.1–220.1 nm. The amplitude difference (ΔA) at the selected wavelengths (ΔA_(214.1 – 220.1 nm)_) were plotted against the relevant concentrations for ASP for calibration curve construction, and the regression equation parameters were calculated.

#### Double divisor-ratio difference spectrophotometric method for DIPH determination

The stored spectra of DIPH were divided by a spectrum of a double divisor (DD) binary mixture of PAR and ASP (15.0 µg/mL, each). The obtained ratio spectra were stored. The difference between the peak amplitudes of ratio spectra at 214.5 nm and 226.0 nm (ΔP_(214.5–226.0 nm)_) *versus* the corresponding concentration was plotted. Calibration curve for DIPH was constructed, and the regression equation was derived.

The absorption spectra of the laboratory prepared mixtures were recorded. Then, the previously mentioned procedure was carried out. By substituting in the respective regression equations, the concentrations of PAR, ASP, and DIPH were determined.

### Application to Excedrin^®^ PM Headache dosage form and content uniformity testing

Ten caplets, each labelled to contain 250 mg PAR, 250 mg ASP, and 38 mg DIPH as citrate salt, were accurately weighed separately and powdered well. An equivalent quantity to one caplet was accurately transferred into a 50-mL volumetric flask with 20-mL methanol, sonicated for 1 h, then completed to volume with methanol and filtered. Appropriate dilution was carried out with methanol so that the concentration of the final prepared solution was 10 µg/mL of PAR, 10 µg/mL of ASP, and 1.5 µg/mL of DIPH. To evaluate the content uniformity of the co-formulated dosage form units, same extraction procedure was followed using one intact caplet. Ten caplets were evaluated separately. The concentration of PAR, ASP, and DIPH were calculated, and the content uniformity was evaluated by the proposed methods.

## Results and discussion

For routine drug analysis, spectrophotometry is a well-established and commonly used technique in quality control laboratories all over the world. Although spectrophotometry seems fair to develop a reasonable, simple, and selective analytical approach compared to the high running cost of the chromatographic techniques, the interferences and overlapping of spectral data from co-formulated pharmaceutical drugs and excipients make direct determination difficult. Consequently, an effective mathematical resolution process is required. Furthermore, spectrophotometry has made major contributions to green analytical chemistry by reducing energy consumption and waste generation.

**Fig. 2:**
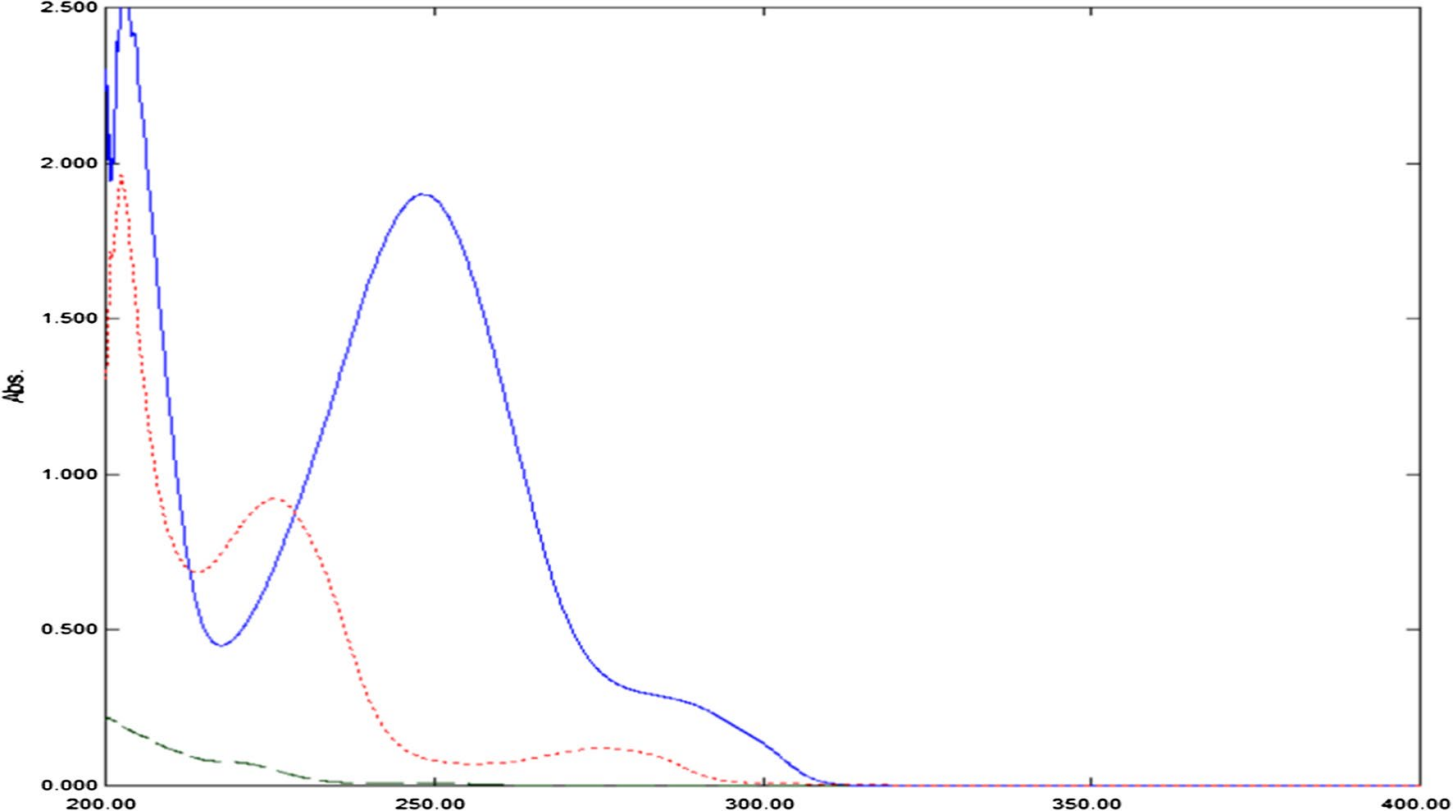
Absorption spectra of 20.0 μg/mL PAR (Blue line), 20.0 μg/mL ASP (red dotted line), and 3.0 μg/mL DIPH (green dashed line) using methanol as blank

After scanning the absorption spectra of the cited drugs against methanol in the wavelength range 200.0–400.0 nm, DIPH shows undefined spectral band at 200.0–235.0 nm, while ASP displays two peaks at 226.0 nm and 276.0 nm. On the other hand, PAR is characterized with λ_max_ at 248.0 nm with spectral extension over ASP and DIPH spectra, Fig. 2. The low absorptivity and hence sensitivity stood against direct assessment of PAR at its expanded region. Furthermore, considerable spectral overlap was observed in the 200.0–300.0 nm region, which could not be resolved by direct spectrophotometry, as shown in Fig. 2. As a result, the major objective was to utilize such spectral characteristics in resolving the overlapped spectral signals of the ternary mixture via developing simple, and smart resolution methods.

### First derivative spectrophotometric method (D^1^)

**Fig. 3:**
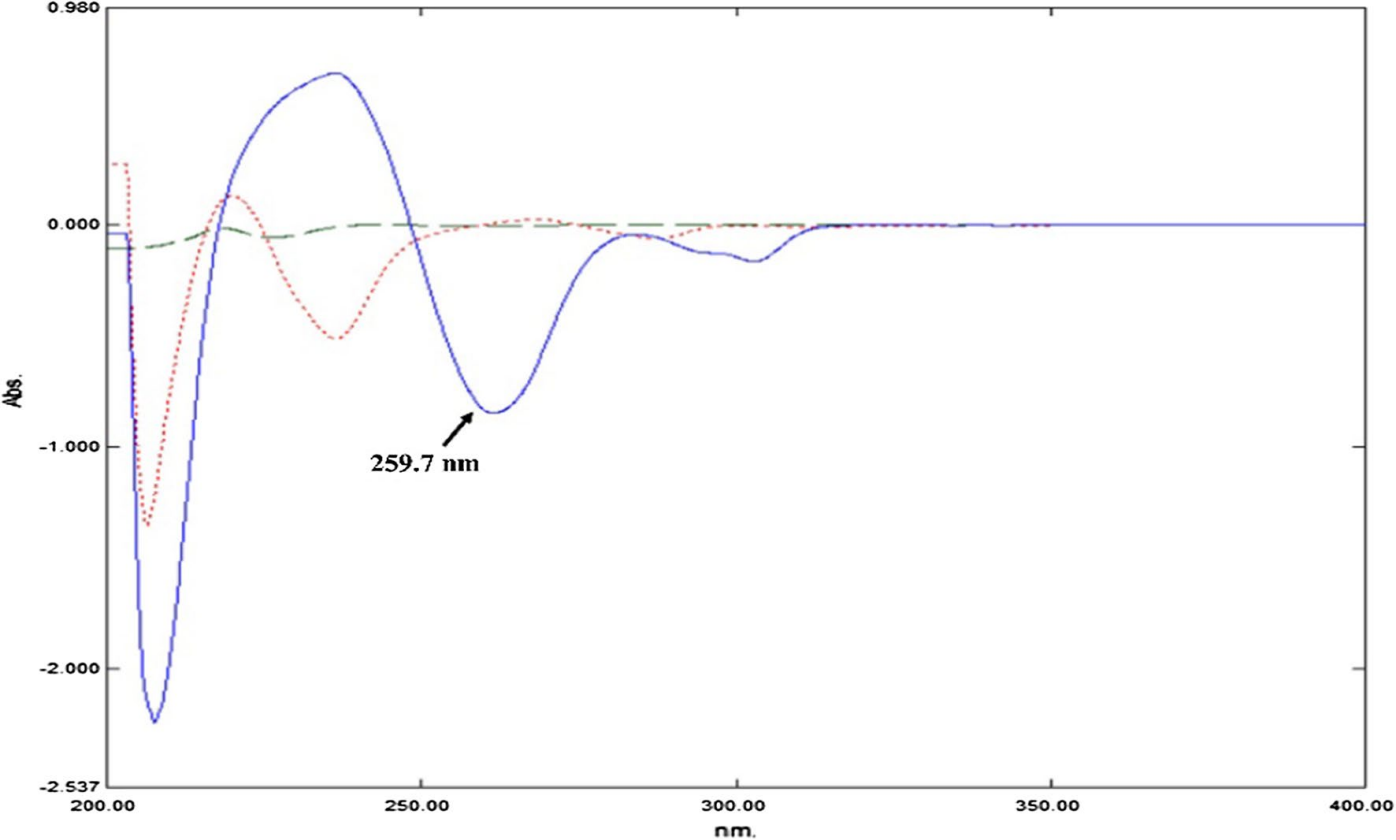
First derivative order (^1^D) absorption spectra of PAR (Blue line), ASP (red dotted line), and DIPH (green dashed line) at concentration 10.0 μg/mL, each

By enhancing the resolution of overlapping bands, first derivative spectrophotometry is a well-established approach for determining components with overlapped spectra in mixtures [17]. The obtained D^1^ spectra of PAR, ASP, and DIPH revealed that PAR could be determined at 259.7 nm, without any interference from ASP or DIPH. As ASP and DIPH showed zero crossing point and zero contribution at this wavelength, respectively, Fig. 3. Calibration curve was constructed and showed a linear relationship between the D^1^ peak amplitude at 259.7 nm and PAR corresponding concentrations.

### Dual wavelength in ratio spectra spectrophotometric method (DWRS)

**Fig. 4:**
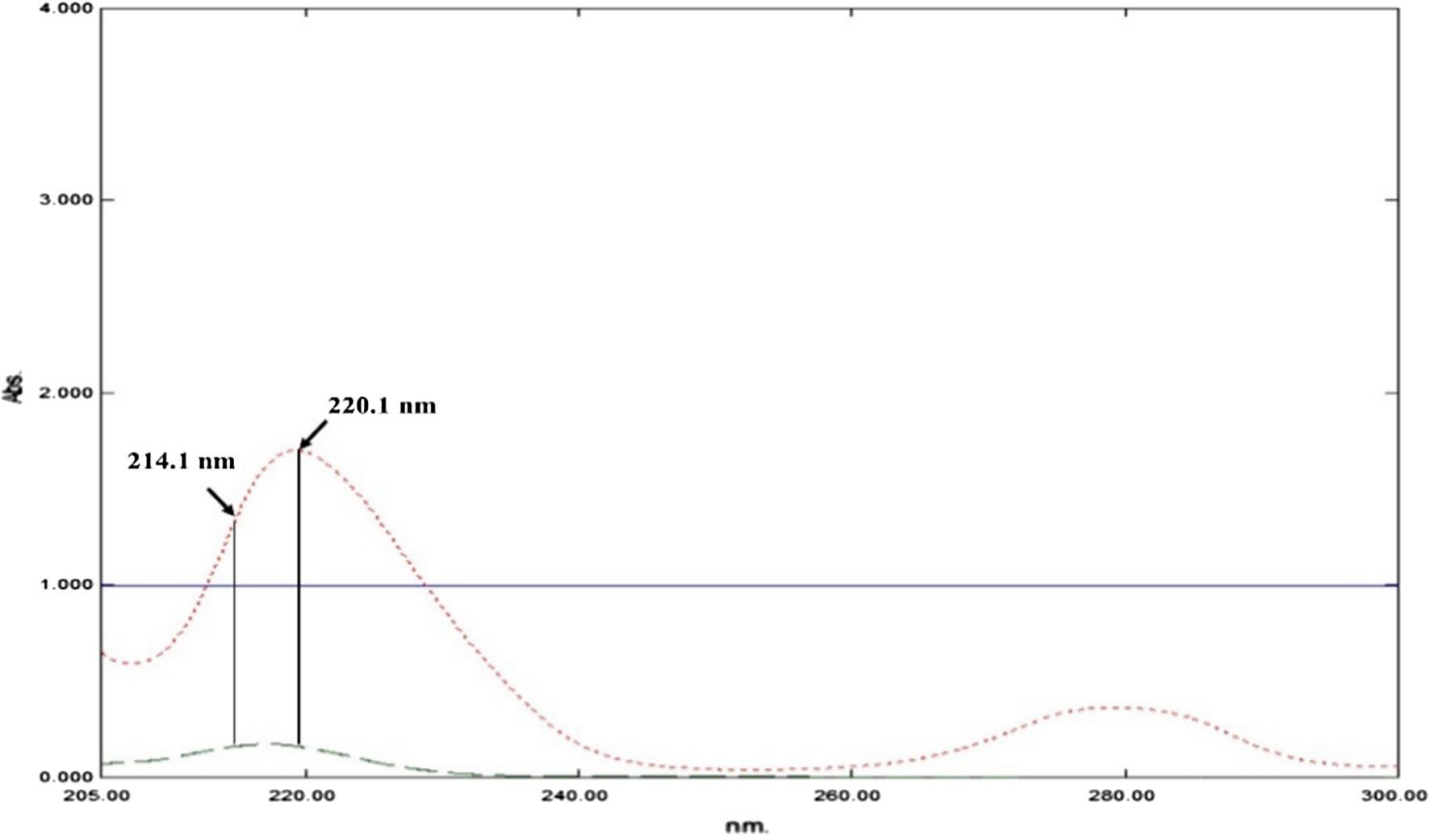
Ratio spectra of PAR (Blue line) 20.0 μg/mL, ASP (red dotted line) 20.0 μg/mL, and DIPH (green dashed line) at 3.0 μg/mL using 10.0 μg/mL PAR as a divisor

By combining the resolving power of two well-known spectrophotometric methods; namely the ratio difference method and dual wavelength, a simple and selective spectrophotometric approach for determining compounds with overlapped spectra in ternary mixtures was developed [18]. The suggested approach can determine compounds in ternary mixtures, where each component may be detected selectively once the other two components' influence has been removed. By using a divisor of the interfering component, the ratio difference method can be used to determine one of two components in a binary mixture at any two wavelengths throughout their linear range in ratio spectra [19, 20]. Whereas, the dual-wavelength method will determine component in the binary mixture, when the two chosen wavelengths have the same absorptivities for the interfering component. The zero-order spectra of the cited drugs were divided by 10.0 µg/mL PAR, producing ratio spectra of the three components, Fig. 4. Two wavelengths were chosen 214.1 and 220.1 nm, where PAR exhibits a straight line with constant standard spectrum amplitudes, while DIPH shows equal amplitudes. Accordingly, the difference of amplitudes at the specified wavelengths ΔA_(214.1–220.1 nm)_ will relate selectively to ASP concentrations.

### Double divisor-ratio difference spectrophotometric method (DD-RD)

**Fig. 5:**
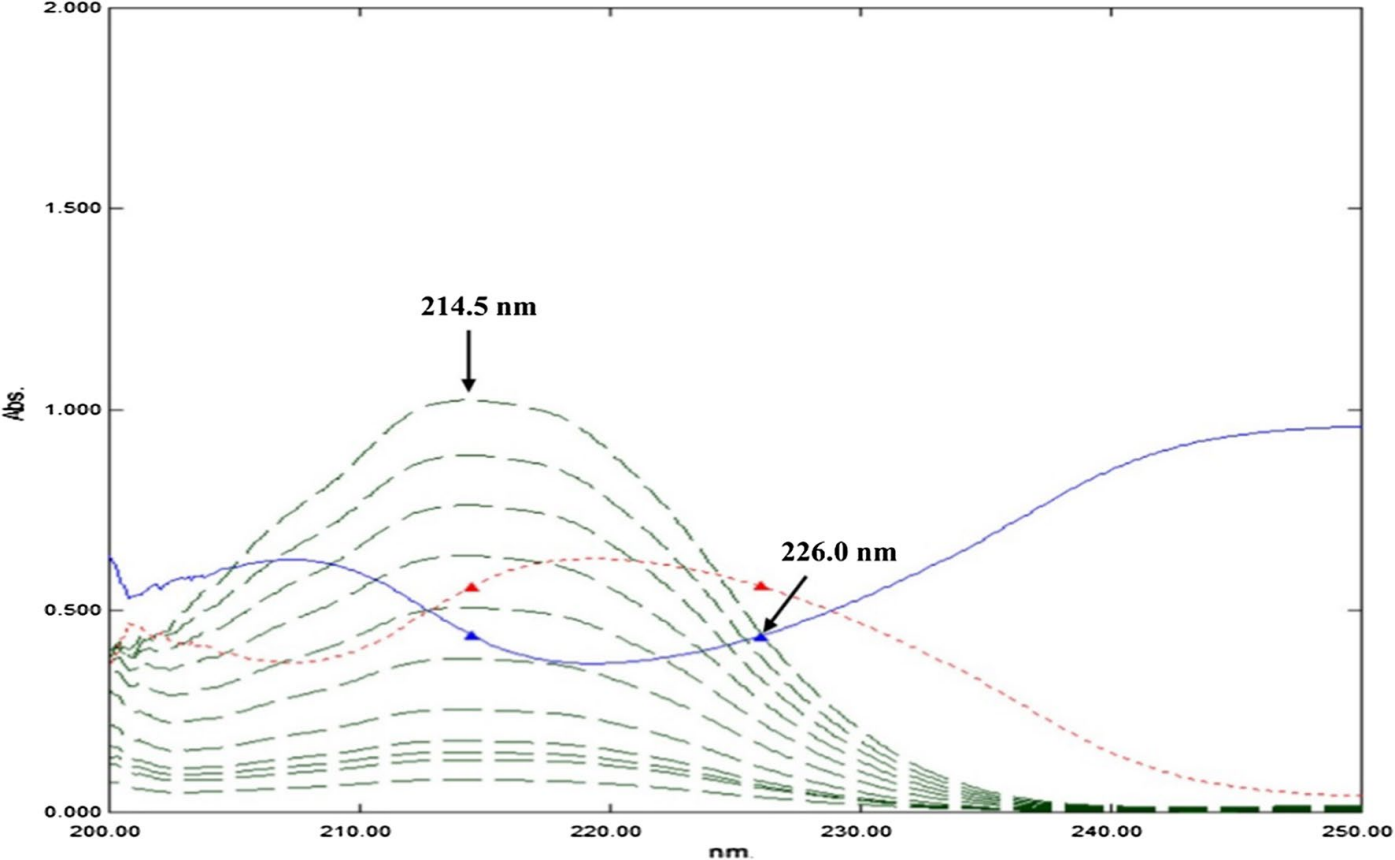
Ratio spectra of DIPH (green dashed line) in the range 1.0–40.0 μg/mL using PAR (Blue line) and ASP (red dotted line) (15.0 μg/mL, each) as a double divisor and methanol as blank

It is a newly established methodology for solving strongly overlapped spectra without prior separation, and it does so without the use of specialized equipment or software [21]. The only prerequisite in evaluating the concentration of the component of interest using the DD-RD approach is the contribution of the three components at the two selected wavelengths λ_1_ and λ_2_, where the amplitudes of the interfering components are the same “constant” in the ratio spectrum, while the component of interest exhibits a considerable difference in these two amplitude values at the two specified wavelengths in respect to concentration [22]. The DD-RD was the most suitable method for determination of DIPH in presence of PAR and ASP. For determination of DIPH, the absorption spectra were divided by a binary mixture spectrum of PAR and ASP, at concentration 15.0 µg/mL, each, generating new ratio spectra. The amplitude at 214.5 and 226.0 nm were chosen and subtracted, so the “constant” for both PAR and ASP will be cancelled, Fig. 5.

## Method validation

Different performance parameters were evaluated according to ICH guidelines in order to ensure the validity of the proposed methods [23]. Working linearity range was established for the proposed methods based on calibration curve construction on three successive days. The concentration ranges of the calibration curves were defined based on the assay of the cited drugs in their dosage form. Small intercept values and the correlation coefficients approaches unity, all indicated acceptable linearity. The linearity ranges and regression equations parameters were illustrated in Table 1.

**Table 1:** Regression parameters and assay validation report of the proposed spectrophotometric methods for determination of pure PAR, ASP and DIPH samples

Parameter	D^1^ method	DWRS method	DD-RD method
	PAR	ASP	DIPH
Wavelength *(λ)*	259.7 nm	Δ*A*_(214.1–220.1 nm)_	Δ*P*_(214.5–226 nm)_
Linearity range (μg/mL)	3.0–40.0	4.0–40.0	1.0–40.0
Slope	0.0398	0.0473	0.0114
SE of slope	0.000216	0.000218	0.000008
Intercept	0.0233	− 0.0459	− 0.0042
SE of intercept	0.004095	0.004203	0.000197
Correlation coefficient *(r)*	0.9999	0.9999	1.000
Accuracy^a^ (Mean ± SD)	100.00 ± 1.04	99.42 ± 1.23	99.48 ± 1.12
Precision^a^			
± (%RSD)^b^	0.83	0.40	0.94
± (%RSD)^c^	1.18	1.67	1.62
LOD (μg/mL)^d^	0.50	0.41	0.19
LOQ (μg/mL)^d^	1.52	1.26	0.57

^a^ Mean and %RSD correspond to the mean and %RSD of the percent recovery

^b^ Intra-day precision [average of 3 different concentrations of 3 replicate each (n = 9) within the same day]

^c^ Inter-day precision [average of 3 different concentrations of 3 replicate each (n = 9) repeated on 3 successive days]

^d^ Limit of detection and quantitation are determined via calculations, LOD = (SD of regression residuals/ slope) × 3.3; LOQ = (SD of regression residuals/ slope) × 10

Accuracy of the developed spectrophotometric methods was checked by analyzing five different concentrations within the established linearity range of the studied drugs in triplicates. The percent recoveries were satisfactory and within the accepted limit, Table 1. Three different concentrations of each cited drug were tested by the proposed methods, in triplicates, within the same day and on three successive days to assess the repeatability and intermediate precision of the procedures, in order. The used concentrations were 7.0, 15.0, and 30.0 µg/mL for PAR, 10.0, 20.0, and 30.0 µg/mL for ASP, and 5.0, 20.0, and 30.0 µg/mL for DIPH. The relative standard deviations (%RSD) obtained were less than two, Table 1.

Furthermore, limit of detection (LOD) and quantification (LOQ) were calculated for the cited drugs using a ratio of 3.3 and 10 standard deviations of the blank and the slope of the calibration curve, respectively. The results for LOD and LOQ are presented in Table 1.

The ability to measure the studied drugs in ternary mixtures with various composition ratios was confirmed by good percent recoveries and %RSD values, Table 2.

**Table 2:** Determination of PAR, ASP and DIPH in synthetic mixtures by the proposed spectrophotometric methods

Ratio (PAR:ASP:DIPH)	Claimed concentration (μg/mL)			% Recovery^a^		
				D^1^ method	DWRS method	DD-RD method
	PAR	ASP	DIPH	PAR	ASP	DIPH
1:1:1	10.0	10.0	10.0	99.42	98.08	100.18
1:1:0.15^b^	10.0	10.0	1.5	99.71	98.58	100.00
4:1:4	20.0	5.0	20.0	101.97	100.17	98.77
3:2:1	15.0	10.0	5.0	101.96	99.56	99.18
1:3:1	5.0	15.0	5.0	101.86	100.01	100.35
Mean ± SD				100.98 ± 1.300	99.28 ± 0.914	99.70 ± 0.684

^a^Average of three determinations

^b^Dosage form ratio

### Analysis of pharmaceutical formulation (Excedrin^®^ PM caplets)

The proposed spectrophotometric methods have been successfully applied to quantify PAR, ASP and DIPH without prior separation or interference from excipients in co-formulated Excedrin^®^ PM caplets. The standard addition technique was further adopted to demonstrate the good performance of the extraction procedure and to ensure the validity of the proposed methods. The results were satisfactory and presented in Table 3.

**Table 3:** Estimation of PAR, ASP and DIPH in Excedrin^®^ PM Headache caplets by the proposed spectrophotometric methods and application of standard addition technique

Drug	Excedrin^®^ PM Headache caplets (BN: 46172679) (Each caplet labelled to contain 250 mg PAR, 250 mg ASP & 38 mg DIPH as citrate salt)	Standard Addition Technique		
	%Found ± SD^a^	Claimed (μg/mL)	Pure added (μg/mL)	%Recovery of the pure added^b^
PAR	100.55 ± 1.320	10	5.0	101.08
			10.0	100.64
			20.0	99.84
		Mean ± SD		100.52 ± 0.631
ASP	100.47 ± 1.507	10	5.0	98.76
			10.0	98.6
			20.0	99.99
		Mean ± SD		99.12 ± 0.760
DIPH	100.16 ± 1.225	1.5	1.0	98.25
			1.5	100.58
			3.0	100.00
		Mean ± SD		99.61 ± 1.217

^a^Average of five determinations

^b^Average of three experiments

### Application on dosage form content uniformity testing

The suggested spectrophotometric methods are well suited for content uniformity evaluation of Excedrin^®^ PM caplets, which is a time-consuming process when applying conventional assay procedures, due to its high precision and ability to immediately estimate the concentration of each drug in a single caplet extract with adequate accuracy. The steps of the test were followed exactly as described by the USP guidelines [24]. The dosage form acceptance value (AV) was calculated using the following equation:

$$AV=\left[M-\overline{X}\right]+KS$$

where, M is a reference value, ($\stackrel{\mathrm{-}}{\text{X}}$) is the mean recovery percent for the assayed caplets, K is the acceptability constant, and S is the standard deviation. Ten caplets were analyzed individually. Each drug of the previously mentioned drugs in their dosage form shows an acceptance value less than the maximum allowable acceptance value (L1) = 15. Therefore, the dosage form content proves the uniformity, as shown in Table 4.

**Table 4:** Results of content uniformity testing of PAR, ASP and DIPH in Excedrin^®^ PM Headache caplets using the proposed spectrophotometric methods

Excedrin^®^ PM Headache caplet No.	% Recovery of the labeled claim		
	PAR D^1^ method	ASP DWRS method	DIPH DD-RD method
1	100.21	99.20	104.46
2	99.21	98.83	101.57
3	99.71	100.88	101.75
4	100.59	99.46	102.10
5	101.85	98.67	101.57
6	102.60	102.00	99.41
7	102.96	101.47	98.68
8	103.23	100.80	101.57
9	99.14	101.58	99.41
10	100.59	99.57	100.61
Mean	101.01	100.24	101.11
SD	1.541	1.234	1.672
%RSD	1.526	1.231	1.654
Acceptance value (AV) ^a^	3.70	2.95	4.01

^a^Maximum allowed Acceptance value (L1) = 15

### Greenness profile evaluation

Green analytical chemistry principles are well-known in chemical laboratories. Specialized evaluation tools are required to accurately analyze the environmental impact of chemical procedures [25]. Because of the importance of environmental protection, there is a growing need to replace traditional pharmaceutical analytical procedures that rely on the usage of dangerous chemicals with more eco-friendly green ones that do not affect performance [26]. Four approaches were introduced to evaluate and grade the eco-friendliness of the proposed methods. We also supplied a detailed benchmark for comparing their environmental impacts. These approaches include the analytical eco-scale, national environmental methods index (NEMI), green analytical procedure index (GAPI), and analytical greenness (AGREE) metric.

The analytical eco-scale approach was used to do a semi-quantitative evaluation by awarding penalty points depending on the influence of various method parameters such as the chemicals used, instrumental energy consumption, generation of waste, and occupational hazards [27]. By subtracting the total parameters penalty points from 100, the ideal green method base value, the analytical eco-score is calculated. The higher the score, the eco-friendlier and more cost-effective the analytical technique. Table 5 shows the eco-scores of the proposed methods and the reported HPLC method [10]. The resulted eco-score indicates the excellent green analysis of the proposed methods.

**Table 5:** Greenness assessment comparison between the proposed spectrophotometric methods and the reported HPLC–DAD method according to Analytical Eco−Scale, NEMI, GAPI and AGREE tools

The proposed spectrophotometric methods				
Eco-Scale assessment		NEMI pictogram	GAPI assessment^c^	AGREE assessment^d^
Reagents	Penalty points (PPs)			
Methanol	6			
Instrument				
Energy consumption^a^	0			
Occupational Hazard	0			
Waste^b^	3			
Total PPs	9			
Analytical Eco-Scale total score	91			
Comment	Excellent green analysis			

The reported HPLC–DAD Method [10]				
Eco-Scale assessment		NEMI pictogram	GAPI assessment^c^	AGREE assessment^d^
Reagents	Penalty points (PPs)			
Methanol	6			
Water	0			
Triethylamine (TEA)	6			
Phosphoric acid (OPA)	2			
Instrument				
Energy consumption^a^	1			
Occupational Hazard	0			
Waste^b^	5			
Total PPs	20			
Analytical Eco-Scale total Score	80			
Comment	Excellent green analysis			

^a^Score of ‘0’ is given as for UV–Vis Spectrometry; < 0.1 kWh per sample and ‘1’ is given as for LC technique; the energy used is ≤ 1.5 kWh per sample

^b^Calculated as: the consumed volume per sample analysis is < 10 mL (sample cuvette volume), while as run time × flow rate for HPLC method

^c^GAPI Assessment evaluated according to Green Analytical Procedure Index parameters description [31]

^d^AGREE Assessment evaluated by using Analytical GREEnness Metric approach and Software [32]

The NEMI is a qualitative approach for assessing the complete environmental safety of an analytical procedure [28]. It employs a straightforward expressive pictogram in the shape of a four-quadrant circle. Each quarter reflects a different criterion, such as the usage of PBT (persistent, bio-accumulative, and toxic) chemicals, hazardous chemical consumption, corrosiveness criteria, and waste generation [29]. According to the US EPA Toxic Release Inventory (TRI) chemical list, methanol is listed in TRI hazardous list but not listed as PBT [30], so the upper right quarter is uncolored. The pH of the solvent is not < 2 or > 12, so the pH is not corrosive. The waste generated per sample is less than 50 g.

The GAPI approach is a new indicator for evaluating the green characteristics of the whole analytical measurements, from sample preparation and collection to the final determination phase [31]. For each phase, a visual pictogram consisting of five pentagrams with a color code ranging from green to yellow to red is used to assign low, medium, and high ecological impact. As shown in Table 5, the proposed methods resulted in more green-covered parts and less red-shaded parts.

The last approach is primarily based on the AGREE software [32]. Based on the 12 principles of Green Analytical Chemistry, AGREE displays a clock-shaped graph with limits separated into 12 sections. To evaluate the analytical procedure's accordance with the green analytical chemistry (GAC) principle, each division corresponds to one principle on a color scale (red-yellow-green). The AGREE graph’s heart contains an overall evaluation color and an overall assessment figure on a scale of 0–1 [32]. The calculator is designed to make procedures more ecologically friendly and safe for the environment. It works by measuring the degree of the greenness of many characteristics such as reagent toxicity, generated waste, energy demands, number of stages, miniaturization, and automation. The parameters used throughout the analysis were each assigned a distinct value, resulting in a final score (0.68) signifying the method's greenness, as shown in Table 5.

## Statistical analysis

Table 6 presents a statistical comparison between the proposed methods and those attained previously using HPLC–DAD one [10]. The calculated Student-t and F values were compared with the theoretical tabulated ones. The comparison revealed that there was no significant difference between the proposed methods and the reported method.

**Table 6:** Statistical comparison of the results obtained by the proposed spectrophotometric methods and the reported HPLC method for the determination of ternary mixture in Excedrin^®^ PM Headache caplets

Parameters	Proposed methods			Reported method [10]^a^		
	PAR	ASP	DIPH	PAR	ASP	DIPH
Mean	100.55	100.47	100.16	99.46	100.15	99.07
SD	1.320	1.507	1.225	1.919	1.751	1.028
n	5	5	5	5	5	5
Variance	1.741	2.271	1.501	3.682	3.066	1.056
Student’s t-test (2.306)^b^	1.046	0.309	1.525	–	–	–
F value (6.39)^b^	2.11	1.35	1.42	–	–	–

^a^HPLC-DAD method was performed using on XTerra C_18_ column with isocratic elution of mobile phase 0.1% triethylamine acidified water: methanol (70:30, v/v) adjusted with *o*-phosphoric acid to pH 3.0 and methanol (90:10, v/v) with flow rate programming and detection at 210.0 nm

^b^Tabulated t- and F values at P = 0.05

## Conclusion

Paracetamol, aspirin, and diphenhydramine, an OTC triple action formula, are frequently used for pain relief, fever control, and night time sleep aid. Currently, this combination is advised for COVID-19 patients as part of symptomatic care. Utilizing spectrophotometric analytical technique for the evaluation and purity verification of pharmaceuticals contributes significantly to the industry's adherence to the requirements of regulatory certification of the drugs and their pharmaceutical products. Accordingly, this work aimed at developing smart, fast, robust and sustainable spectrophotometric methods to resolve the completely overlapped spectra of an over-the-counter ternary mixture of PAR, ASP and DIPH, for the first time. The distinctive spectral features of the studied mixture boosts the development of three univariate spectrophotometric methods, namely; first derivative spectrophotometry for determination of PAR, dual-wavelength in ratio spectra for determination of ASP using PAR as a divisor and double divisor-ratio difference spectrophotometric one based on using the sum of PAR and ASP as a double divisor for determination of DIPH. The proposed methodologies' greenness profile was thoroughly examined through the most prevalent and advanced indexes; analytical eco-scale system, NEMI, GAPI, and AGREE and compared with the reported conventional chromatographic one. The suggested methods exhibit pronounced eco-friendly, simplicity, reliability, accuracy, and selectivity for assaying the studied drugs in pure form and their co-formulated pharmaceutical dosage form. They can be regarded as useful and favourable for routine pharmaceutical quality control studies without preliminary separation. Furthermore, they do not require any sophisticated instruments, lengthy sample preparation steps promoting their applicability in content uniformity testing of the commercially available products. They are suitable for use in research labs lacking conventional liquid chromatographic systems.

## Data Availability

All data generated or analysed during this study are included in this published article.
